# Efficacy of the polo-like kinase inhibitor rigosertib, alone or in combination with Abelson tyrosine kinase inhibitors, against break point cluster region-c-Abelson-positive leukemia cells

**DOI:** 10.18632/oncotarget.4047

**Published:** 2015-05-08

**Authors:** Seiichi Okabe, Tetsuzo Tauchi, Yuko Tanaka, Juri Sakuta, Kazuma Ohyashiki

**Affiliations:** ^1^ Department of Hematology, Tokyo Medical University, Tokyo, Japan

**Keywords:** chronic myeloid leukemia, ABL tyrosine kinase inhibitor, resistant cell, polo-like kinase

## Abstract

The potency of Abelson (ABL) tyrosine kinase inhibitors (TKIs) against chronic myeloid leukemia (CML) has been demonstrated. However, ABL TKI resistance can develop. In this study, we investigated the efficacy of a combination therapy including rigosertib (ON 01910.Na), a polo-like kinase (PLK) and phosphoinositide 3-kinase (PI3K) inhibitor, and ABL TKIs. A 72-h rigosertib treatment was found to inhibit cell growth, induce apoptosis, reduce phosphorylation of the breakpoint cluster region-c (BCR)-ABL and its substrate Crk-L, and increase the activities of caspase 3 and poly (ADP-ribose) polymerase (PARP). This combination therapy also exerted a synergistic inhibitory effect on Philadelphia chromosome (Ph)-positive cell proliferation and reduced the phosphorylation of BCR-ABL and Crk-L while increasing that of cleaved PARP and the H2A.X histone. Rigosertib also potently inhibited the growth of ABL TKI-resistant cells, and cotreatment with ABL TKIs and rigosertib induced higher cytotoxicity. These results indicate that rigosertib treatment may be a powerful strategy against ABL TKI-resistant cells and could enhance the cytotoxic effects of ABL TKIs.

## INTRODUCTION

Chronic myeloid leukemia (CML) is associated with the Philadelphia chromosome (Ph), which is a translocation of the Abelson (*ABL*)1 oncogene on chromosome 9 with a breakpoint cluster region (BCR) on chromosome 22 and is designated t(9;22) [1]. This fusion gene encodes the chimeric oncogenic fusion protein BCR-ABL, a constitutively active tyrosine kinase. Imatinib, an orally available ABL tyrosine kinase inhibitor (TKI), effectively induces cytogenic remission in patients with CML [2]. Despite the efficacy of imatinib, some patients develop resistance to ABL TKI therapy. Primary or secondary resistance to imatinib has been reported to occur in approximately 40% of patients with CML in the chronic phase (CML-CP) [3]. Second-generation ABL TKIs such as nilotinib or dasatinib have been shown to induce major cytogenetic responses in approximately 50% of patients with imatinib-resistant or intolerant CML [4, 5] and are currently used clinically as first-line therapies for newly diagnosed CML [6, 7].

Although the majority of patients with CML-CP achieve impressive clinical responses, increasing evidence of acquired resistance to TKIs has been documented [8]. In particular, the T315I mutation seems to be the most frequently observed resistance mechanism after dasatinib treatment [9]. The TKI ponatinib is also effective for patients with heavily pretreated resistant CML and for one third of patients with accelerated phase (AP) or blastic phase (BP) CML [10]. In the Ph-positive ALL and CML Evaluation (PACE) trial, ponatinib inhibited the growth of leukemia cells, including those harboring T315I mutants [11]. Although ponatinib was developed as a BCR-ABL inhibitor, recent studies have identified novel compound mutations that cause resistance to ponatinib [12, 13], indicating that mutations in the BCR-ABL kinase domain may confer resistance to ABL TKIs. Moreover, ABL TKIs cannot eradicate leukemia stem cells [14]. Therefore, alternative strategies are needed to improve the outcomes of patients with CML.

Mitosis is the key event of cell cycle, and polo-like kinase (PLK) is a pivotal regulator of mitosis as well as cytokinesis [15]. Five members of the PLK family have been discovered in humans. PLK1, which is mainly expressed during the late G2 and M phase, regulates various stage of mitosis. This protein contains a highly conserved N-terminal protein kinase domain and a C-terminal polo box domain (PBD), which is required for docking to other targets. PLK1 is also highly expressed in a broad spectrum of tumors, and its expression level correlates with poor prognosis in patients. Therefore, high PLK1 levels are a marker of cellular proliferation in cancers such as leukemia [16, 17].

Rigosertib, also known as ON0190.Na, is a benzyl sulfone analogue and an adenosine triphosphate (ATP)-noncompetitive, multitargeted inhibitor [18]. Rigosertib inhibits both PLK1 and phosphatidylinositol 3-kinase (PI3K) and has been investigated in clinical trials against solid tumors and hematological malignancies such as myelodysplastic syndrome (MDS) [19, 20]. Herein, we have investigated the effects of rigosertib against Ph-positive leukemia cells. We have also investigated whether co-treatment with ABL TKIs and rigosertib would increase cytotoxicity against Ph-positive primary leukemia cells.

## RESULTS

### Rigosertib induces cytotoxicity and inhibits the proliferation of Ph-positive cell lines

**Figure 1:**
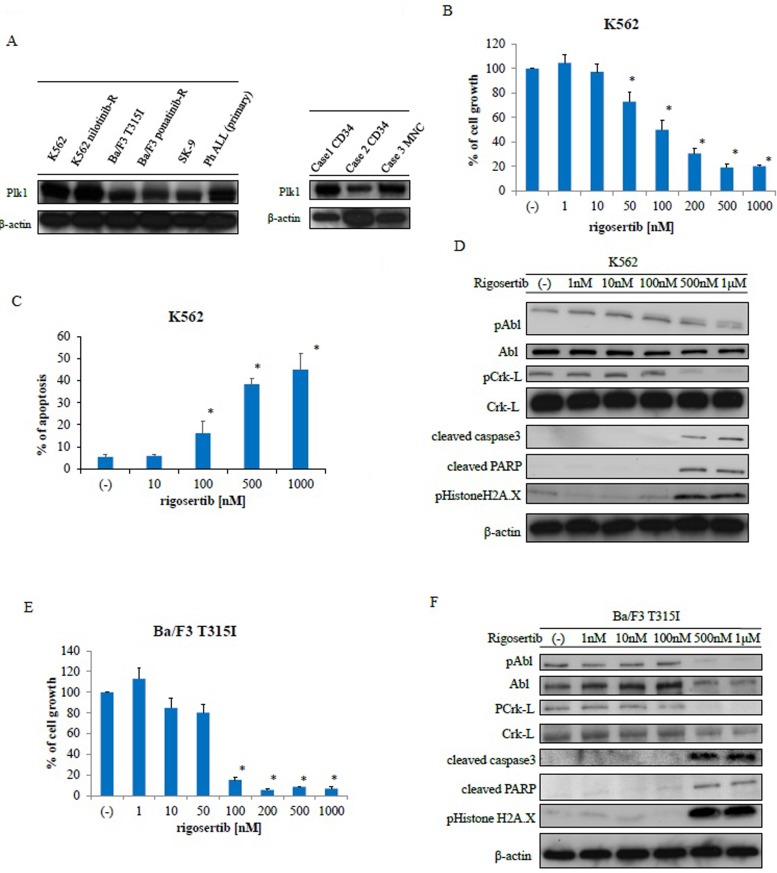
Analysis of polo-like kinase 1 expression and rigosertib activity against Philadelphia (Ph)-positive leukemia cells **A.** Polo-like kinase 1 (PLK1) expression was examined via immunoblotting, with actin as the loading control. **B.**, **E.** K562 or Ba/F3 T315I cells were treated with the indicated concentrations of rigosertib for 72 h. The percentages of cell growth were examined; *P < 0.05 compared with the control. Results represent three separate experiments. **C.** K562 cells were treated with the indicated concentrations of rigosertib for 48 h. Percentages of apoptotic cells were examined. These results represent three independent experiments; *P < 0.05 compared with the control. **D.**, **F.** K562 or Ba/F3 T315I cells were treated with rigosertib at the indicated concentrations for 24 h. Total extracts were examined via immunoblotting with anti-phospho ABL, phospho-Crk-L, phosphohistone H2A.X, cleaved caspase 3, cleaved-PARP, ABL, Crk-L, and β-actin antibodies (abs). ABL, Abelson; PARP, poly (ADP-ribose) polymerase; abs, antibodies.

PLK1 is up-regulated in many human tumors, including hematological malignancies [17]. Therefore, we first examined PLK1 expression in Ph-positive leukemia cells and observed expression in Ph-positive leukemia cell lines and primary samples as well as cluster of differentiation (CD) 34-positive CML samples (Figure 1A). As rigosertib is a potent PLK1 and PI3K inhibitor, we examined its efficacy against Ph-positive leukemia cells. Incubation for 72 h with the indicated concentrations of rigosertib decreased growth (Figure 1B) and increased apoptosis in K562 cells in a dose-dependent manner (Figure 1C). Immunoblotting revealed reduced phosphorylation of BCR-ABL and its downstream substrate Crk-L but increased caspase 3 and PARP activity after rigosertib treatment (Figure 1D). We also observed increased H2A.X histone phosphorylation after treatment with rigosertib. Patients with resistance to imatinib therapy harbor the T315I mutation [8], and we, therefore, evaluated the efficacy of rigosertib against T315I-mutant Ba/F3 cells. Cell growth was inhibited in a dose-dependent manner after a 72-h treatment with rigosertib (Figure 1E). In an immunoblot analysis, rigosertib treatment reduced the phosphorylation of BCR-ABL and Crk-L and increased both the caspase and PARP activity levels, as well as H2A.X phosphorylation (Figure 1F).

### Co-treatment with ABL TKIs and rigosertib increased cytotoxicity against Ph-positive cell lines

**Figure 2:**
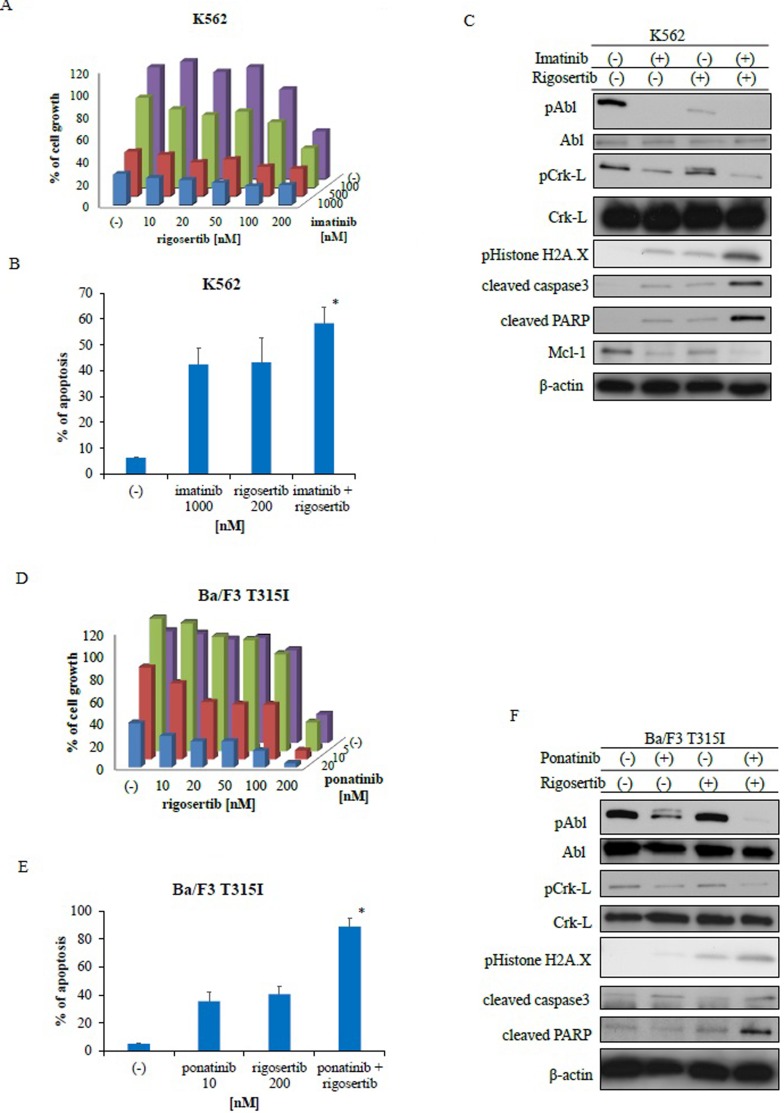
Co-treatment with Abelson tyrosine kinase inhibitors and rigosertib inhibits the proliferation of Philadelphia (Ph)-positive leukemia cells **A.**, **D.** K562 or Ba/F3 T315I cells were treated with the indicated concentrations of rigosertib or imatinib, both, or ponatinib for 72 h. The percentages of cell growth were determined as described in the Methods. Data are representative of three independent sets of experiments. **B.**, **E.** K562 or Ba/F3 T315I cells were treated with the indicated concentrations of ponatinib or rigosertib, both, or imatinib for 48 h. Percentages of apoptotic cells was determined; *P* < 0.05 compared with imatinib or ponatinib treatment. **C.**, **F.** K562 or Ba/F3 T315I cells were treated with ponatinib or rigosertib, both, or imatinib for 24 h. Total cellular lysates were analyzed via immunoblotting with anti-phospho ABL, phospho-Crk-L, phosphohistone H2A.X, cleaved caspase 3, cleaved-PARP, ABL, Crk-L, Mcl-1 and β-actin antibodies. ABL, Abelson; PARP, poly (ADP-ribose) polymerase; Mcl-1, myeloid cell leukemia.

We tested the effect of a combination of rigosertib (0, 10, 20, 50, 100, and 200 nM) and imatinib (0, 100, 500 nM, and 1 μM) on Ph-positive K562 cells. The combination of the two drugs decreased the proliferation of K562 cells more than each drug alone (Figure 2A). Additionally, combined treatment with imatinib and rigosertib led to apoptosis at a higher rate than with each drug alone (Figure 2B). Immunoblot analysis revealed that phosphorylation of BCR-ABL and Crk-L decreased whereas caspase 3 and PARP activities increased following co-treatment with imatinib and rigosertib (Figure 2C) and that H2A.X phosphorylation also increased. Furthermore, the combination of imatinib and rigosertib down-regulated the expression of the anti-apoptotic protein myeloid cell leukemia (Mcl)-1. T315I mutant cells are resistant to second-generation ABL TKIs. We found that co-treatment with ponatinib and rigosertib decreased the proliferation of T315I mutant cells (Figure 2D). A two-way analysis of variance (ANOVA) revealed that these drugs acted in a synergistic manner (Figure 2A, 2D). Combined treatment with ponatinib and rigosertib also increased apoptosis in T315I mutant cells (Figure 2E), and immunoblotting revealed decreased phosphorylation of BCR-ABL and Crk-L and increased caspase 3 and PARP activities (Figure 2F) along with increased H2A.X phosphorylation. These results suggest that rigosertib enhances ABL TKI-induced cytotoxicity against Ph-positive leukemia cells.

### Rigosertib overcomes ABL TKI-resistant leukemia cells

**Figure 3:**
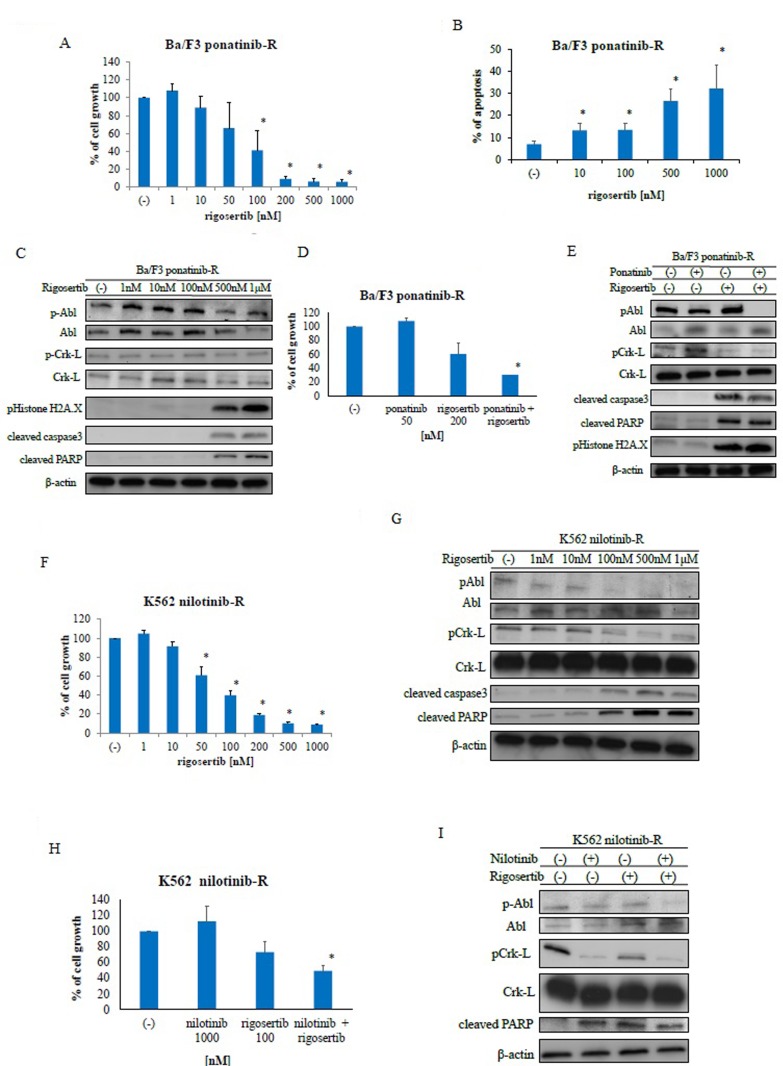
Rigosertib activity against Abelson tyrosine kinase inhibitor -resistant cells **A.**, **F.** Cells were cultured in the presence or absence of rigosertib for 72 h. Viable cell numbers were counted; *P < 0.05 compared with the control. Results represent three independent experiments. **B.** Cells were treated with the indicated concentrations of rigosertib for 48 h. Percentages of apoptotic cells were determined; *P < 0.05 compared with the control. **C.**, **G.** Cells were treated with rigosertib at the indicated concentrations for 24 h. Total extracts were examined via immunoblotting with anti-phospho ABL, phospho-Crk-L, phosphohistone H2A.X, cleaved caspase 3, cleaved-PARP, ABL, Crk-L, and β-actin antibodies. **D.**, **H.** Cells were treated with the indicated concentrations of rigosertib or ponatinib, both, or nilotinib for 72 h. Percentages of cell growth were determined; *P < 0.05 compared with rigosertib treatment. **E.**, **I.** Cells were treated with nilotinib or rigosertib, both, or ponatinib for 24 h. Total extracts were examined via immunoblotting with anti-phospho ABL, phospho-Crk-L, phosphohistone H2A.X, cleaved caspase 3, cleaved-PARP, ABL, Crk-L, and β-actin antibodies. ABL, Abelson; PARP, poly (ADP-ribose) polymerase.

To investigate whether rigosertib could sensitize ABL TKI-resistant cells to ABL TKIs, a cell growth assay was performed using Ba/F3 ponatinib-resistant (Ba/F3 ponatinib-R) cells and nilotinib-resistant K562 (K562 nilotinib-R) cells. We found that treatment with rigosertib reduced the growth of these cells in a dose-dependent manner (Figure 3A). We also observed increased apoptosis (Figure 3B) and activation of caspase 3 and PARP (Figure 3C) in Ba/F3 ponatinib-R cells following rigosertib treatment; in contrast, rigosertib reduced ABL and Crk-L phosphorylation in a dose-dependent manner. Further, we investigated the efficacy of ponatinib and rigosertib against ponatinib-resistant Ba/F3 cells. Although ponatinib treatment did not increase the activity of PARP, co-treatment with ponatinib and rigosertib inhibited cell growth (Figure 3D) and increased PARP cleavage, caspase 3 activity, and H2A.X phosphorylation (Figure 3E). We further examined the efficacy of rigosertib against K562 nilotinib-R cells and found that this agent decreased cell growth in a dose-dependent manner (Figure 3F). Rigosertib treatment also reduced the phosphorylation of BCR-ABL and Crk-L while increasing the activities of caspase 3 and PARP (Figure 3G). We further observed increased cytotoxicity and reduced phosphorylation of BCR-ABL and Crk-L following co-treatment with nilotinib and rigosertib (Figures 3H, 3I). These results indicate that rigosertib increases the cytotoxicity of ABL TKIs against ABL TKI-resistant cells.

### PLK1 knockdown induces cytotoxicity against Ph-positive leukemia cells

**Figure 4:**
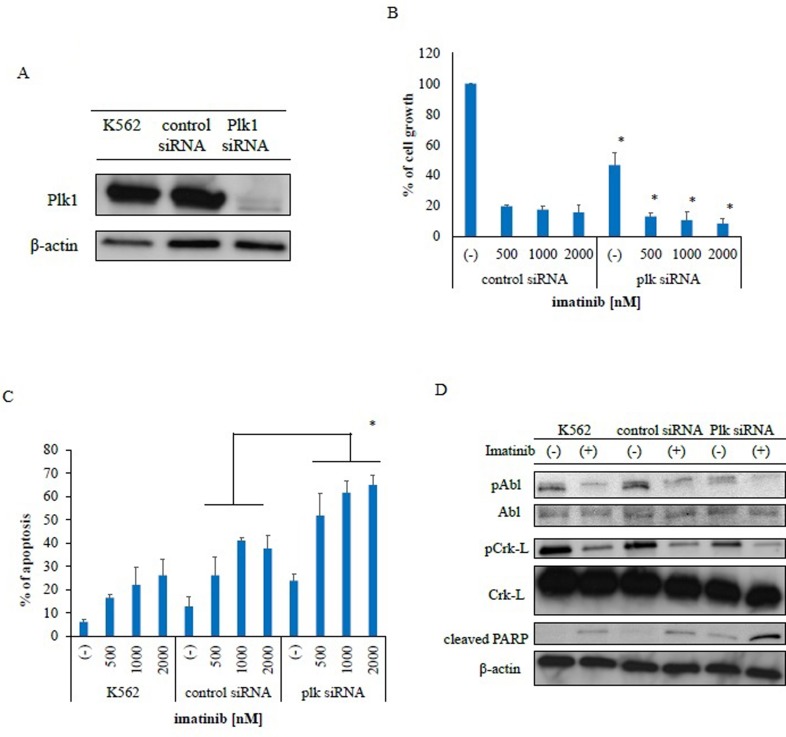
Transfection of polo-like kinase 1-specific siRNA inhibits proliferation and promotes apoptosis in Philadelphia (Ph)-positive leukemia cells **A.** PLK1 protein expression was measured via immunoblotting with a PLK1-specific antibody. β-actin was used as a loading control. **B.** PLK1 and control siRNA transfected cells were incubated with imatinib for 72 h. Viable cell numbers were counted; *P < 0.05 compared with control siRNA transfected cells. These results represent three separate experiments. **C.** Cells were treated with imatinib for 48 h. Percentages of apoptotic cells were determined; *P* < 0.05 compared with control siRNA transfected cells. Results represent three independent experiments. **D.** Cells were treated with imatinib for 24 h. Whole extracts were examined via immunoblotting with anti-phospho ABL, phospho-Crk-L, cleaved-PARP, and β-actin antibodies. ABL, Abelson; PARP, poly (ADP-ribose) polymerase.

We investigated the inhibitory effects of PLK1 on Ph-positive leukemia cells by examining the effects of PLK1 siRNA knockdown in K562 cells. Immunoblot analysis revealed that PLK1 expression was completely inhibited (Figure 4A). Subsequently, we observed inhibited growth inhibition in these PLK1 siRNA-transfected cells (Figure 4B) and greater imatinib-mediated inhibition in the PLK1 siRNA-transfected K562 cells relative to the control siRNA-transfected cells. We also observed increased apoptosis in the PLK1 siRNA-transfected cells following treatment with imatinib (Figure 4C). Immunoblot analysis revealed increased PARP activity following imatinib treatment in the PLK1 siRNA-transfected K562 cells (Figure 4D). These results indicate that PLK1 knockdown increased the sensitivity of these cells to ABL TKI.

### Rigosertib is effective against Ph-positive primary tumor samples

**Figure 5:**
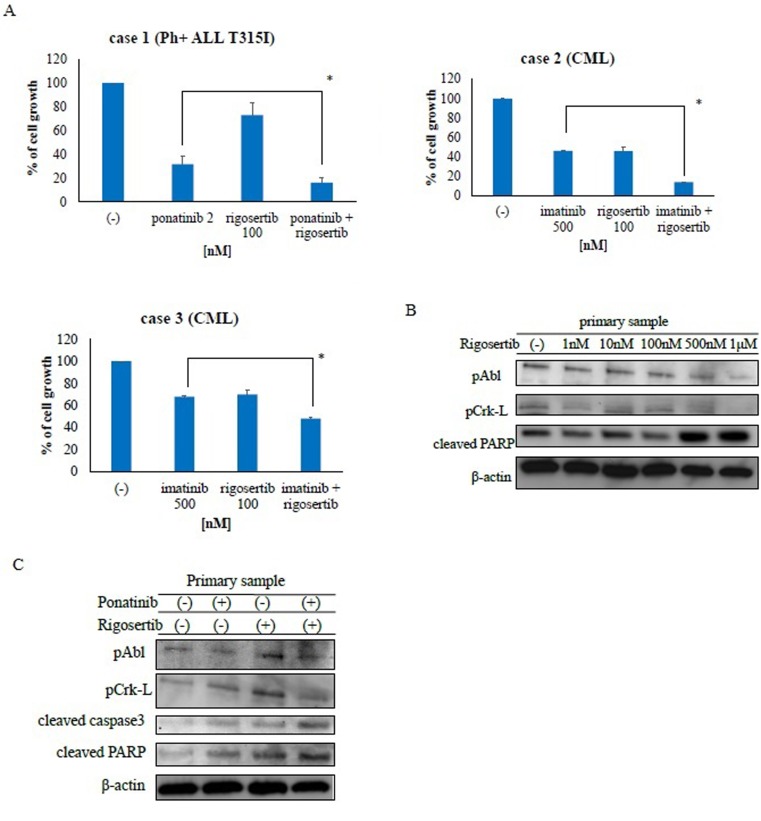
Rigosertib suppresses growth and alters signaling in Philadelphia (Ph)-positive primary cells **A.** CML or Ph-positive ALL cells were treated with ponatinib or rigosertib, both, or imatinib for 72 h. Cell viability was determined as described in the Methods; *P < 0.05 compared with ponatinib or imatinib treatment. **B.** Lysates from primary cells treated with or without rigosertib for 24 h were immunoblotted using the indicated antibodies. **C.** Cells were treated with ponatinib, rigosertib, or both for 24 h. Whole extracts were examined via immunoblotting with anti-phospho ABL, phospho-Crk-L, cleaved caspase 3, cleaved-PARP and β-actin antibodies. CML, chronic myeloid leukemia; ALL, acute lymphoblastic leukemia; PARP, poly (ADP-ribose) polymerase.

The activity of rigosertib has been investigated in numerous types of cancer cells [20]. Accordingly, we examined the activity of rigosertib against Ph-positive primary tumor samples. Co-treatment with ABL TKIs and rigosertib strongly inhibited cell proliferation relative to treatment with each drug alone (Figure 5A). Next, we examined intracellular signaling mechanisms and found that rigosertib treatment increased PARP activity (Figure 5B) while inhibiting the phosphorylation of BCR-ABL and Crk-L. Co-treatment with ponatinib and rigosertib also increased PARP activity in primary T315I-positive cells while decreasing the phosphorylation of BCR-ABL and Crk-L (Figure 5C). These results indicate that co-treatment with ABL TKI and rigosertib enhanced the cytotoxicity against Ph-positive primary samples.

## DISCUSSION

Recent studies have suggested PLK1 as a useful target in the treatment of various tumors. PLK1 is the most well studied member of the PLK family and a key regulator of mitotic progression [15]. In this study, we demonstrated that PLK1 protein was abundantly expressed in Ph-positive leukemia and CD34-positive primary CML cells. These observations support the use of PLK1 as a therapeutic target.

Because PLK1 expression is increased in CD34-positive CML stem cells (Figure 1A), PLK inhibition may eradicate these cells. We demonstrated that one PLK1 inhibitor, rigosertib, could induce H2A.X phosphorylation in Ph-positive leukemia cells (Figures 1D, 1F). H2A.X phosphorylation is a sensitive cellular response to various events that results in DNA damage [21]. Therefore, rigosertib may induce an early chromatin modification event following DNA damage and fragmentation. Our data also demonstrated that PLK1 inhibition synergistically enhanced sensitivity to ABL TKIs in Ph-positive cells. In cancer cells, PLK1 depletion has been shown to inhibit cell proliferation [22], perturb spindle assembly, prolong mitotic arrest, and induce apoptosis. In this study, we also confirmed that siRNA-induced PLK1 depletion inhibited cell growth, increased apoptosis, and enhanced the efficacy of ABL TKI (Figure 4C).

Rigosertib is a small molecule that induces mitotic arrest, and we showed that rigosertib alone could induce dose-dependent cytotoxicity in ABL TKI-resistant cell lines. In contrast, no response was observed in ABL TKI-resistant cells even after 72 h of treatment with ABL TKIs such as nilotinib and ponatinib, when administered alone. Other studies have reported that rigosertib alone induces apoptosis in hematological cancer cells [23]. We demonstrated that co-treatment with ABL TKIs and rigosertib inhibited cell proliferation, induced apoptosis, activated caspase-3, and altered the expression of Mcl-1 (Figure 2C).

ABL TKI-resistant patients have a poor prognosis. Accordingly, we investigated the efficacy of rigosertib against ABL TKI-resistant cell lines. Rigosertib is currently being investigated in clinical trials and has shown promising results when administered alone or as part of a combination therapy [24]. Other PLK inhibitors have also been investigated [25]. PLK1 inhibition by a combination of rigosertib and ABL TKIs offers a novel targeted anti-tumor therapy for patients with Ph-positive leukemia.

In summary, this pre-clinical study provides scientific rationale for the continued investigation of PLK inhibition as a therapeutic strategy for patients with Ph-positive leukemia. We also provide new information regarding the efficacy of combined treatment with the PLK inhibitor rigosertib and a potent ABL TKI against ABL TKI-resistant leukemia cells.

## MATERIALS AND METHODS

### Ethics statement

Peripheral blood samples were collected from patients with CML and Ph-positive leukemia after obtaining written informed consent. The Institutional Review Board of the Tokyo Medical University approved this study in adherence to the Declaration of Helsinki.

### Ph-positive cell lines and patient cells

The Ph-positive leukemia cell line (K562) was obtained from the American Type Culture Collection (ATCC, Manassas, VA, USA). K562 nilotinib-R [26], a T315I mutant Ph-positive ALL cell line (SK-9) [27], Ba/F3 ponatinib-R [13], and T315I mutant Ba/F3 cells [28] were established previously. All cell lines were cultured in Roswell Park Memorial Institute (RPMI) 1640 medium containing 10% fetal bovine serum (FBS) and maintained at 37°C in a 5% CO_2_ humidified atmosphere. Fresh peripheral blood samples were collected in heparinized tubes from patients after obtaining informed consent. Mononuclear cells were separated from blood using LymphoSepare (Immuno-Biological Laboratories, Minneapolis, MN, USA). These cells were used immediately or cryopreserved in liquid nitrogen until used.

### Reagents

Rigosertib and ponatinib were purchased from MedKoo Biosciences (Chapel Hill, NC, USA), and imatinib and nilotinib were provided by Novartis Pharma AG (Basel, Switzerland). Stock solutions of rigosertib, nilotinib, and ponatinib were prepared in dimethyl sulfoxide (DMSO). Imatinib was dissolved in distilled water, aliquoted, and stored at −20°C. Anti-phospho ABL, phospho Crk-L, cleaved caspase 3, and PARP antibodies (Abs) were purchased from Cell Signaling (Danvers, MA, USA). Anti-phosphohistone H2A.X and Crk-L Abs were obtained from Millipore (Billerica, MA, USA). ABL, PLK1, and Mcl-1 Abs were obtained from Santa Cruz Biotechnology (Santa Cruz, CA, USA). Other reagents were obtained from Sigma chemical company (St. Louis, MO, USA).

### siRNA transfection

PLK1-targeting siRNA was purchased from Santa Cruz Biotechnology (sc-36277). K562 cells were transfected with PLK1 or control siRNA via electroporation as described previously [29].

### Cell viability assays

The viability of Ph-positive cells treated with rigosertib alone or in combination with imatinib, nilotinib, or ponatinib was measured via trypan blue exclusion or staining with a cell counting kit solution (Dojin, Kumamoto, Japan) followed by photometric measurements at A450 nm to determine cell viability. The experiments were performed in triplicate.

### Apoptosis assay

Apoptosis of the Ph-positive cell lines was assayed using previously described methods [30]. The experiments were performed in triplicate.

### Immunoblotting

Immunoblot analysis was performed according to previously described methods [31]. Ph-positive cell lines or patient cells were incubated with the indicated concentrations of ABL TKI or rigosertib for the indicated time periods. Following the indicated treatments, cells were harvested and lysed by sonication in radioimmunoprecipitation assay (RIPA) lysis buffer. Protein lysate concentrations were measured using a protein assay kit (Bio-Rad Laboratories, Hercules, CA, USA). Equal amounts of proteins were loaded on 4%–20% polyacrylamide gel and transferred to polyvinylidene difluoride (PVDF) membranes. The membranes were probed with the primary antibodies of interest at the appropriate dilutions for 2 h at room temperature. Blots were developed using an Amersham enhanced chemiluminescence (ECL) plus kit (GE Healthcare, Little Chalfont, UK). The experiments with Ph-positive cell lines were performed thrice independently.

### Statistical analysis

Differences in dose responses and apoptosis between treatment groups were determined using Student's *t* test. *P* values of <0.05 were considered significant. In some experiments, results of multiple-group comparisons were analyzed using a two-way ANOVA and presented as means ± standard deviations.
